# Changes of Plasma B-Type Natriuretic Peptide Levels after High-Pressure Post-Dilation following Coronary Stent Deployment

**DOI:** 10.1371/journal.pone.0082357

**Published:** 2013-12-06

**Authors:** Gang-Yong Wu, Gang-Jun Zong, Jing-Kai Chen, Yang Xia, Man-Qing Chen, Xiao Wang, Xiao-Ying Wang, Lu-Lu Wang, Tian-Xiao Wang

**Affiliations:** Department of Cardiology, No. 101 Hospital of PLA, Wuxi, Jiangsu Province, China; University of Louisville, United States of America

## Abstract

**Objective:**

To evaluate the changes of plasma B-type natriuretic peptide(BNP) levels after high-pressure post-dilation following coronary stent deployment.

**Methods:**

A total of 173 patients undergoing percutaneous coronary intervention for the left anterior descending artery were enrolled into the study. All patients were divided into two groups: the conventional group and the post-dilation group. The plasma BNP, troponin I(TnI), myocardial band isoenzyme of creatine kinase(CK-MB) levels and the serum high sensitive C-reactive protein(hs-CRP) levels immediately before and 24 hours after the interventional procedures were compared between the two groups.

**Results:**

There were no significant differences between the two groups in terms of clinical features, clinical and biochemical parameters, stent parameters, pre-procedural plasma BNP and TnI levels, pre-procedural serum hs-CRP levels, as well as pre- and post-procedural CK-MB levels (all *P*>0.05). In the conventional group, post-procedural plasma BNP levels were significantly reduced when compared with the pre-procedural levels, median(25th,75th) were 32.5 ng/L(15.0,52.4) vs. 37.7 ng/L(18.2,67.3), *P* = 0.001. In the post-dilation group, post-procedural plasma BNP levels were significantly increased when compared with the pre-procedural levels, median(25th,75th) were 53.5 ng/L(29.6,82.8) vs. 44.2 ng/L(17.15,70.7), *P*<0.0001. Post-procedural plasma TnI levels were also significantly increased when compared with the pre-procedural levels in both groups, median(25th,75th) were 0.02 ng/L(0.01,0.08) vs. 0.01 ng/L(0.01,0.01), 0.05 ng/L(0.01,0.35) vs. 0.01 ng/L(0.01,0.01), respectively, *P*<0.0001, so were the serum hs-CRP levels, median(25th,75th) were 3.3 mg/L(2.4,4.7) vs. 2.2 mg/L(1.4,3.3), 4.2 mg/L(3.175,5.825) vs. 2.3 mg/L(1.45,3.6), respectively, *P*<0.0001. Post-procedural plasma BNP, TnI and serum hs-CRP levels in the post-dilation group were significantly higher than those in the conventional group(all *P*<0.0001).

**Conclusion:**

High-pressure post-dilation following coronary stent deployment resulted in a significant increase of plasma BNP levels, as well as plasma TnI levels and serum hs-CRP levels, which may be related to myocardial perfusion, more myocardial injury and more inflammation.

## Introduction

With the advances in cardiovascular interventional technology and materials, percutaneous coronary intervention plays an increasingly important role in the treatment of coronary heart diseases. The optimal deployment of coronary stent is the most important step to ensure a successful percutaneous coronary intervention. In daily clinical practice, high-pressure post-dilation with a non-compliant balloon is frequently used to achieve complete stent apposition. Previous studies have shown that post-dilation can improve the stent expansion and reduce the risk of stent thrombosis. This technology has been widely used in coronary interventional procedures [1],[2],[3],[4]. However, we found that in our clinical practice, some patients undergoing high-pressure balloon post-dilation suffered from some symptoms such as mild chest discomfort or short of breath shortly after the procedure. Whether such symptoms meant cardiac dysfunction was uncertain. With regard to the relationship between the cardiac function and high-pressure post-dilation, it remained unclear due to the lack of related studies. On the one hand, the use of post-dilation technology may induce plaque over-compression and increase plaque debris. On the other hand, high-pressure post-dilation may result in uneven force balance within the stent and even stent fracture, as well as uneven plaque compression with a greater degree of plaque rupture. The plaque debris may affect the myocardial blood supply after reaching distal coronary microvascular, thereby affecting the cardiac function. A correct evaluation of the relationship between post-dilation and cardiac function will help guide the use of high-pressure balloon post-dilation in clinical practice.

Plasma B-type natriuretic peptide is synthesized by the myocardial cells, containing a 32-amino acid peptide that is expressed within the ventricles. It is rapidly synthesized and released into the circulation in response to increased ventricular wall pressure or stretching. B-type natriuretic peptide is a quantitative index of heart failure, which is very valuable in highly accurate diagnosis and prognosis assessment of the heart failure, and has been included in current guidelines for chronic heart failure management[5]. Therefore, we chose plasma B-type natriuretic peptide as the assessed parameter of cardiac function in all enrolled patients.

## Materials and Methods

### Patients

From June 2007 to December 2011, a total of 580 patients underwent percutaneous coronary intervention in our hospital. After excluding those with acute myocardial infarction, chronic occlusive lesions, major complications such as coronary perforation, dissection, major bleeding, and contrast-induced nephropathy during or after the original interventional procedures, 173 cases undergoing single sirolimus-eluting stent implantation in left anterior descending(LAD) artery were enrolled into this study and retrospectively analyzed. All interventional procedures for the enrolled patients were performed by same interventional cardiologists, without any cardiovascular complications during hospital stay. All enrolled patients were divided into two groups according to interventional methods: a conventional-pressure deployment group (conventional group, n = 79) and a non-compliant balloon high-pressure post-dilation group (post-dilation group, n = 94).

### Research Methods

All general information in both groups was analyzed, including age, gender, body mass index (dividing the weight by square of height,kg/m2), total cholesterol(TC), high density lipoprotein cholesterol(HDL-C), low density lipoprotein cholesterol(LDL-C), triglyceride(TG), fasting plasma glucose(FPG), blood pressure immediately before and 24 hours after procedure. Hypertension, dyslipidemia, diabetes mellitus, smoking and alcohol drinking were recorded by yes/no questionnaire[6],[7],[8].

All procedural stent parameters in both groups were analyzed, including: 1. comparing the stent length, stent diameter, stent deployment pressure during percutaneous coronary intervention between the two groups; 2. evaluating the noncompliant balloon diameter and post-dilation pressure in the post-dilation group.

All procedural imaging data in both groups were analyzed. According to the imaging data, the indication of high-pressure post-dilation for selected patients was assessed by at least two experienced interventional cardiologists.

The plasma B-type natriuretic peptide, troponin I, myocardial band isoenzyme of creatine kinase levels and the serum high sensitive C-reactive protein levels were assessed for all cases in both groups immediately before and 24 hours after the coronary intervention. The pre- and post-procedural plasma B-type natriuretic peptide levels were tested to assess the impact of high-pressure post-dilation on post-procedural plasma B-type natriuretic peptide, thereby to clarify whether the high-pressure post-dilation technology has an impact on cardiac function shortly after the interventional procedure. B-type natriuretic peptide level was tested with a immunofluorescence method (Triage B-type natriuretic peptide Test, Biosite Inc, San. Diego, CA, USA), troponin I levels were tested with a direct chemiluminescence method (troponin assays, SIEMENS, USA),and myocardial band isoenzyme of creatine kinase levels were tested with a immunosuppression method(myocardial band isoenzyme of creatine kinase assays, KANTO CHEMICAL CO.,INC. JAPAN).

### Ethics Statement

The Medical Ethics Committee of No. 101 Hospital of PLA approved this retrospective study(NO. 20120205). Written consents were given by the patients for their information to be stored in the hospital database and used for research.

### Statistical Analysis

Categorical variables were presented as frequencies(%). Shapiro-Wilk test was used to determine whether the distribution of continuous variables was normal. Normally distributed variables were presented as means and standard deviation, using Student's t test, whereas non-normally distributed variables were presented as medians(25th,75th percentiles), using non-parametric analysis. Moreover, Wilcoxon signed-rank test was performed to compare the difference between the 2 time points. All statistical assessments were 2-sided and evaluated at the 0.05 level of significant difference. Statistical analyses were performed using SPSS 15.0 statistical software (SPSS Inc, Chicago, III).

## Results

### General information of enrolled patients in both groups

As shown in Table 1 and Table 2, there were no significant differences in terms of clinical features including age, gender, hypertension, dyslipidemia, diabetes mellitus, smoking and alcohol drinking between the two groups, as well as clinical and biochemical parameters including body mass index, pre- and post-procedural blood pressure, TC, LDL-C, HDL-C, TG and FPG (all *P*>0.05).

**Table 1:** Clinical Features of Patients in both groups.

	Conventional Group (n:79)	Post-dilation Group (n:94)	*P*
Age(yrs)	62.5±6.4	63.7±5.5	0.483
Gender(male/female)	46/33	51/43	0.646
Hypertension(yes/no)	25/54	32/62	0.749
Diabetes(yes/no)	11/68	14/80	1.000
Dyslipidemia(yes/no)	3/76	5/89	0.729
Smoking(yes/no)	39/40	42/52	0.545
Alcohol(yes/no)	10/69	15/79	0.665

**Table 2:** Clinical and Biochemical Parameters of Patients in both groups(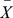 ±S).

	Conventional Group (n:79)	Post-dilation Group (n:94)	*P*
Body mass index(kg/m^2^)	25.6±2.3	25.7±2.8	0.483
FPG(mmol/L)	5.81±1.63	6.32±2.07	0.121
TG(mmol/L)	1.91±0.4	1.92±0.3	0.385
LDL-C(mmol/L)	2.45±0.8	2.47±0.9	0.935
HDL-C(mmol/L)	1.12±0.3	1.21±0.3	0.390
TC(mmol/L)	5.45±0.9	5.48±0.8	0.615
Before procedure			
SBP(mmHg)	140.2±26.8	145.4±21.3	0.085
DBP(mmHg)	80.4±15.6	81.8±14.2	0.235
24 hours after procedure			
SBP(mmHg)	141.8±23.2	148.4±25.8	0.072
DBP(mmHg)	82.5±12.7	83.6±14.6	0.965

FPG:fasting plasma glucose; TG:triglyceride; LDL-C:low density lipoprotein-cholesterol; HDL-C:high density lipoprotein-cholesterol; TC:total cholesterol; SBP:systolic blood pressure; DBP:diastolic blood pressure.

TC,LDL-C,HDL-C: mmol/L = mg/dl×0.0259; TG: mmol/L = mg/dl×0.0113; mmHg = 0.1333 Kpa.

### Procedural device parameters of enrolled patients in both groups

As shown in Table 3, there were no significant differences in terms of stent diameter, stent length and deployment pressure between the two groups (all *P*>0.05).

**Table 3:** Procedural device Parameters of Patients in both groups.

	Conventional Group (n:79)	Post-dilation Group (n:94)	*P*
Stent length (mm)	21.78±7.70	22.46±7.10	0.748
Stent diameter (mm)	3.27±0.37	3.28±0.39	0.968
Deployment pressure (atm)	14.50±1.50	13.18±3.91	0.148
Post-dilation pressure (atm)	-	20.5±3.9	

atm: atmosphere.

In the post-dilation group, the mean post-dilation pressure was (20.5±3.9) atm.

### Procedural imaging data of enrolled patients in both groups

**Figure 1:**
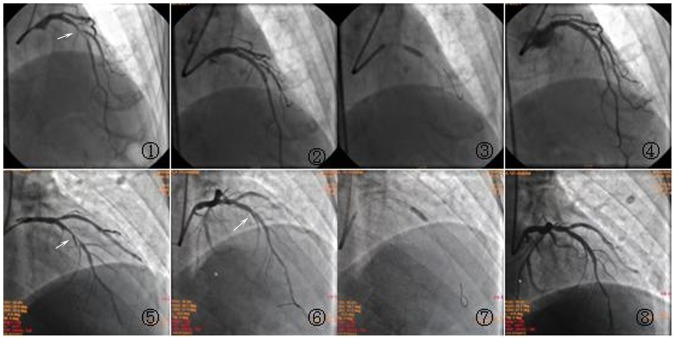
High-pressure post-dilation following coronary stent deployment. 1. Long lesions at the middle of LAD with 90% stenosis, the vessel diameter difference was significant between the proximal and distal segment. 2. Stent deployment in the LAD lesions. 3. Proximal stented segment was post-dilated with a non-compliant balloon. 4. Stent well-deployment after post-dilation,the proximal and distal segment morphology was even. 5. Tubular lesions at the middle of LAD with 90% stenosis,the vessel diameter difference was not significant between the proximal and distal segment. 6. Stent deployment in the LAD lesions and the middle of stent was incomplete apposition. 7. Middle stented segment was post-dilated with a non-compliant balloon. 8. Stent well-deployment after post-dilation,the proximal and distal segment morphology was even.

In all enrolled patients, high-pressure post-dilation was used in the following two situations: 1, If the stent length was ≥24 mm and the target vessel diameter difference was significant between the proximal and distal segments, proximal stented segment would be routinely post-dilated using a high-pressure balloon; 2, If the diameter difference was not significant between the proximal and distal segments, high-pressure balloon post-dilation was only used in patients with incomplete stent apposition (Figure 1). Procedural imaging data was analyzed by two experienced interventional cardiologists.

### Changes of plasma B-type natriuretic peptide, troponin I, myocardial band isoenzyme of creatine kinase levels and serum high sensitive C-reactive protein levels before and after interventional procedures in both groups

In the conventional group, post-procedural plasma B-type natriuretic peptide levels were significantly reduced when compared with the pre-procedural levels, median(25th,75th) were 32.5 ng/L(15.0,52.4) vs. 37.7 ng/L(18.2,67.3), *P* = 0.001. However, post-procedural plasma troponin I and serum high sensitive C-reactive protein levels were significantly increased when compared with the pre-procedural levels, median(25th,75th) were 0.02 ng/L(0.01,0.08) vs. 0.01 ng/L(0.01,0.01), 3.3 mg/L(2.4,4.7) vs. 2.2 mg/L(1.4,3.3), respectively, *P*<0.0001(Table 4). There was no significant difference between the plasma myocardial band isoenzyme of creatine kinase levels before and after the interventional procedure ([15.4±5.3]U/L vs. [15.5±4.7]U/L, *P*>0.05).

**Table 4:** Pre-post procedural levels of BNP, TnI and hs-CRP in conventional group(M(Q1,Q3)).

Index	Pre-procedural	Post-procedural	*P*
BNP(ng/L)	37.7(18.2,67.3)	32.5(15.0,52.4)	0.001
TnI(ng/L)	0.01(0.01,0.01)	0.02(0.01,0.08)	<0.0001
hs-CRP(mg/L)	2.2(1.4,3.3)	3.3(2.4,4.7)	<0.0001

BNP: B-type natriuretic peptide; TnI:troponin I; hs-CRP:high sensitive C-reactive protein. (the same below)

In the post-dilation group, however, post-procedural plasma B-type natriuretic peptide levels were significantly increased when compared with the pre-procedural levels, median(25th,75th) were 53.5 ng/L(29.6,82.8) vs. 44.2 ng/L(17.15,70.7), *P*<0.0001. Post-procedural plasma troponin I and high sensitive C-reactive protein levels were as well significantly increased when compared with the pre-procedural levels, median(25th,75th) were 3.3 ng/L(2.4,4.7) vs. 2.2 ng/L(1.4,3.3), 4.2 mg/L(3.175,5.825) vs. 2.3 mg/L(1.45,3.6), respectively, *P*<0.0001(Table 5). There was no significant difference between the plasma myocardial band isoenzyme of creatine kinase levels before and after the interventional procedure([16.2±4.8]U/L vs. [16.5±5.7]U/L, *P*> 0.05).

**Table 5:** Pre-post procedural levels of BNP, TnI and hs-CRP in post-dilation group(M(Q1,Q3)).

Index	Pre-procedural	Post-procedural	*P*
BNP(ng/L)	44.2(17.15,70.7)	53.5(29.6,82.8)	<0.0001
TnI(ng/L)	0.01(0.01,0.01)	0.05(0.01,0.35)	<0.0001
hs-CRP(mg/L)	2.3(1.45,3.6)	4.2(3.175,5.825)	<0.0001

Pre-procedural plasma B-type natriuretic peptide, troponin I, myocardial band isoenzyme of creatine kinase levels and serum high sensitive C-reactive protein levels were similar between the two groups(Table 6), as well as post-procedural plasma myocardial band isoenzyme of creatine kinase levels(all *P*>0.05). However, post-procedural plasma B-type natriuretic peptide, troponin I levels and serum high sensitive C-reactive protein levels in the post-dilation group were significantly higher than those in the conventional group(all *P*<0.0001) (Table 7).

**Table 6:** Pre-procedural levels of BNP, TnI and hs-CRP between both groups (M(Q1,Q3)).

Index	Conventional group	Post-dilation Group	*P*
BNP(ng/L)	37.7(18.2,67.3)	44.2(17.15,70.7)	0.528
TnI(ng/L)	0.01(0.01,0.01)	0.01(0.01,0.01)	0.397
hs-CRP(mg/L)	2.2(1.4,3.3)	2.3(1.45,3.6)	0.336

**Table 7:** Post-procedural levels of BNP, TnI and hs-CRP between both groups (M(Q1,Q3)).

Index	Conventional group	Post-dilation Group	*P*
BNP(ng/L)	32.5(15.0,52.4)	53.5(29.6,82.8)	<0.0001
TnI(ng/L)	0.02(0.01,0.08)	0.05(0.01,0.35)	<0.0001
hs-CRP(mg/L)	3.3(2.4,4.7)	4.2(3.175,5.825)	<0.0001

## Discussion

Coronary stent implantation has become the preferred strategy for reconstruction of stenotic arteries and restoration of distal myocardial blood supply. Post-procedural lumen area is an important assessment index for successful interventional procedures. However, stent under-deployment frequently occurs after stent implantation, due to lumen stenosis and/or plaque calcification. In this case, adequate post-dilation is often needed. Especially after the advent of intravascular ultrasound, high-pressure balloon post-dilation has been increasingly used in clinical practice. Previous studies have shown that high-pressure post-dilation is helpful for optimal stent deployment, uniform stent expansion, and improved stent apposition, thereby increasing the minimum stent area, decreasing the rates of stent thrombosis and restenosis, and reducing the long-term risks of target lesion revascularization and major adverse cardiac events[9].

However, the high-pressure post-dilation is not a perfect technology and may result in stent deformation or fracture, leading to increased late restenosis rate[10]. In addition, some reports showed that among patients with acute myocardial infarction, post-dilation was associated with a significantly higher risk of death/myocardial infarction but not associated with the risk of repeat revascularization. Whereas, among patients who did not present with acute myocardial infarction, post-dilation was not associated with risks of death/myocardial infarction or repeat revascularization[11],[12]. However, no data are currently available to clarify whether the high-pressure post-dilation has an adverse effect on short-term cardiac function in patients without acute myocardial infarction.

In the results from this study, there was no significant difference in pre-procedural general information such as clinical features and biochemical parameters between the two groups. Post-procedural myocardial band isoenzyme of creatine kinase levels were also similar to the pre-procedural levels in both groups, which did not support the diagnosis of percutaneous coronary intervention related myocardial infarction. However, post-procedural plasma troponin I levels were higher when compared with pre-procedural levels in both groups, which may be related to the minor myocardial injury, inflammatory response and the high sensitivity of troponin I. During the interventional procedure, plaque debris may affect the micro-circulatory system after reaching distal coronary microvascular, leading to minor myocardial injury and eventuall leading to elevated troponin I levels.

C-reactive protein is an acute phase protein synthesized by the liver and is considered to be one of the most sensitive indicators of inflammation. In the results from this study, post-procedural serum high sensitive C-reactive protein levels in post-dilation group were significantly increased when compared with the levels in conventional group, suggesting that the inflammatory reaction in post-dilation group was stronger. Obviously, it was related to excessive squeeze on the plaque and endothelial damage following high-pressure post-dilation.

In the conventional group, plasma B-type natriuretic peptide levels at 24 hours after procedure were significantly reduced compared with those before the procedure, and the difference achieved statistical significance, suggesting that distal blood flow was improved and ventricular wall pressure was decreased after target vessel revascularization. In the post-dilation group, plasma B-type natriuretic peptide levels at 24 hours after procedure were significantly increased compared with those before the procedure, and the difference also achieved statistical significance. Conventional-pressure dilation provides a uniform force on the vessel wall and the plaques become thin and apposite, showing a consistency of shape changes. After stent implantation, the lumen area is expanded, the distal microcirculation and the ischemic myocardial perfusion are improved, and thus the myocardial function is meliorated. High-pressure post-dilation often results in uneven force balance in the stent, regional plaque rupture following high-pressure compression, and micro-thrombus formation which may cause distal embolization, affect the micro-circulatory system, and lead to stunning of myocardial function. In addition to myocardial perfusion, we found that the post-procedural plasma troponin I levels and serum high sensitive C-reactive protein levels in the post-dilation group were significantly higher than those in the conventional group, thus we conjecture that more myocardial injury and more inflammation were involved. Myocardial injury and inflammation would result in decreased myocardial function, leading to elevated B-type natriuretic peptide levels. As we know, any levels of B-type natriuretic peptide under 100 ng/L rules out heart failure while levels of B-type natriuretic peptide exceed 400 ng/L is one of diagnostic criteria for heart failure, and in this study, though the differences between pre- and post-procedual in both groups are significant, the absolute values are too small to draw the the conclusion that the difference in B-type natriuretic peptide reflects a change in cardiac function, that is to say, whether the high-pressure post-dilation has an adverse effect on short-term cardiac function in patients with coronary stent deployment is uncertain.

The present study results remind the interventional cardiologists that they should pay more attention to adverse reactions such as myocardial microcirculation disfunction, excessive inflammation and tissue trauma in patients undergoing high-pressure post-dilation in coronary interventions. For those with heart failure before the percutaneous coronary intervention, B-type natriuretic peptide should be carefully monitored if the post-dilation is necessary, especially at the early period after intervention. Some therapies can be given in advance to avoid or relieve these adverse reactions.

The main limitations of this study included retrospective study design, small sample size, a few parameters, short observation period, and relatively simple endpoints. In the present study, we only enrolled patients undergoing percutaneous coronary intervention of single-vessel disease of left anterior descending artery. Especially, some objective means of corroboration to cardiac dysfunction like imaging with cardiac ultrasound or physiological data such as a documented increase in left ventricular filling pressures are absent. Therefore, the results can only provide a trend, and the cause analysis is only a theoretical speculation. Further studies are needed to clarify the detailed mechanism for B-type natriuretic peptide increase shortly after high-pressure post-dilation and changes in cardiac function after interventional treatment of multi-vessel diseases.
